# An evaluation of inexpensive methods for root image acquisition when using rhizotrons

**DOI:** 10.1186/s13007-017-0160-z

**Published:** 2017-03-07

**Authors:** Awaz Mohamed, Yogan Monnier, Zhun Mao, Guillaume Lobet, Jean-Luc Maeght, Merlin Ramel, Alexia Stokes

**Affiliations:** 1 AMAP, INRA, CNRS, IRD, Université de Montpellier, Montpellier, France; 2 Earth and Life Institute, Université catholique de Louvain, 1348 Louvain-la-Neuve, Belgium; 3 Agrosphere-IBG3, Forschungszentrum Juelich GmbH, 52428 Juelich, Germany; 4 iEES-Paris (IRD, Sorbonne Universités, UPMC Univ Paris 06, CNRS, INRA, UPEC, Université Paris Diderot), Soils and Fertilizers Research Institute (SFRI), Duc Thang Ward, Bac Tu Liem District, IRD, Hanoi, Vietnam

**Keywords:** Fine root elongation rate, Flatbed scanner, Handheld scanner, Smartphone, Time-lapse camera

## Abstract

**Background:**

Belowground processes play an essential role in ecosystem nutrient cycling and the global carbon budget cycle. Quantifying fine root growth is crucial to the understanding of ecosystem structure and function and in predicting how ecosystems respond to climate variability. A better understanding of root system growth is necessary, but choosing the best method of observation is complex, especially in the natural soil environment. Here, we compare five methods of root image acquisition using inexpensive technology that is currently available on the market: flatbed scanner, handheld scanner, manual tracing, a smartphone application scanner and a time-lapse camera. Using the five methods, root elongation rate (RER) was measured for three months, on roots of hybrid walnut (*Juglans nigra* × *Juglans regia* L.) in rhizotrons installed in agroforests.

**Results:**

When all methods were compared together, there were no significant differences in relative cumulative root length. However, the time-lapse camera and the manual tracing method significantly overestimated the relative mean diameter of roots compared to the three scanning methods. The smartphone scanning application was found to perform best overall when considering image quality and ease of use in the field. The automatic time-lapse camera was useful for measuring RER over several months without any human intervention.

**Conclusion:**

Our results show that inexpensive scanning and automated methods provide correct measurements of root elongation and length (but not diameter when using the time-lapse camera). These methods are capable of detecting fine roots to a diameter of 0.1 mm and can therefore be selected by the user depending on the data required.

## Background

Fine root growth, defined as apical elongation over time [1–3], plays an essential role in the cycling and allocation of carbon and nutrients in ecosystems [4]. Due to the inaccessibility of root systems, special techniques are required to investigate the distribution and dynamics of roots, as well as to estimate belowground carbon budgets [5, 6]. Today, a number of methods have been used to estimate root growth. These methods can be grouped into indirect and direct techniques [5, 7], both of which have advantages and drawbacks. Indirect methods include the use of empirical models [8], estimations of nitrogen budget and carbon budget [7]. Direct methods can be classified into (i) destructive techniques such as soil coring [9], sequential soil coring [5], in-growth cores [5, 10], monoliths [11–13] and soil pits [14–16], and (ii) nondestructive in situ methods including isotope quantification [17], ‘root windows’ or rhizotrons [18–21] and minirhizotrons [3, 22, 23]. Although there are several criticisms concerning these techniques [17], rhizotrons and minirhizotrons are considered as efficient approaches and are commonly used to characterize fine root growth [24–26]. Rhizotrons can be used to monitor (from initiation to mortality) specific root segments at frequent time intervals without significantly impacting root processes [15]. However, the drawbacks of these techniques are related to the cost of installation and potential changes in soil hydrology and physics, which would affect estimates of root production [7, 27].

Although many studies on root growth using minirhizotrons have been performed [23, 28], only a small part of the root system can be observed. Techniques for observing root growth include recording root images with digital cameras [23, 29] and rotating scanners (CID, Inc., WA, USA) [3], but equipment is expensive. Results from contrasting methods on one single species can also be highly variable [15]. The disparity in results obtained from different methods [15, 16, 30–33] has also been attributed to differences in the software used for image analysis [34–36].

The quality of images obtained from minirhizotrons and rhizotrons is extremely important for an accurate quantification of root growth through image analysis. The advantage of rhizotrons over minirhizotrons, is that a variety of inexpensive techniques exist for quantifying root growth in the field. Root systems can be measured by tracing onto a transparent plastic sheet [21], scanning with a flatbed scanner [30, 32, 34], or a handheld scanner [37]. Scanners have often been considered as the most useful tool for obtaining high quality images, but necessitate the use of a power supply in the field and are not yet fully automated. A detailed comparison of the different types of scanners available has also not yet been performed, especially with regard to the scanners now available as digital applications on smartphones and tablets. Such a comparative study would be highly useful, especially when choosing a particular scanner for a given application and considering its cost, robustness, automation and the quality of the images produced.

With regard to recording automatically images in the field, systems that are independent of an electrical power supply are not yet available, although automated flatbed scanners using 12v batteries for several days have been tested successfully in a teak (*Tectona grandis* ***)*** plantation in Lao PDR (Maeght, unpublished data). A fully automated method for measuring root elongation in the field would permit studies of growth in poorly accessible areas or with a poor power supply, as well as detailed measurements of e.g. effects of the circadian clock on root growth in situ [38]. To date, most circadian clock studies have been performed in the laboratory on young plants [38, 39]. Therefore, the necessity of developing a fully automated technique to measure root growth in the field is of major importance.

We compared the quality of images obtained, and the advantages or disadvantages when using several different types of scanner to measure root growth in the field. We focused on inexpensive technology that is currently available on the market and so is accessible to a wide range of potential users. We assessed these scanning techniques in conjunction with a fully manual method (tracing onto a plastic sheet) and a fully automated method (time-lapse camera). Measurements were performed in hybrid walnut (*Juglans nigra* × *regia* L.) agroforests in France. Results are discussed with regard to quality, time, and cost criteria.

## Methods

### Comparison of methods for acquiring images

We examined five methods for acquiring images of root systems growing in rhizotrons:

#### Flatbed scanner

**Fig. 1:**
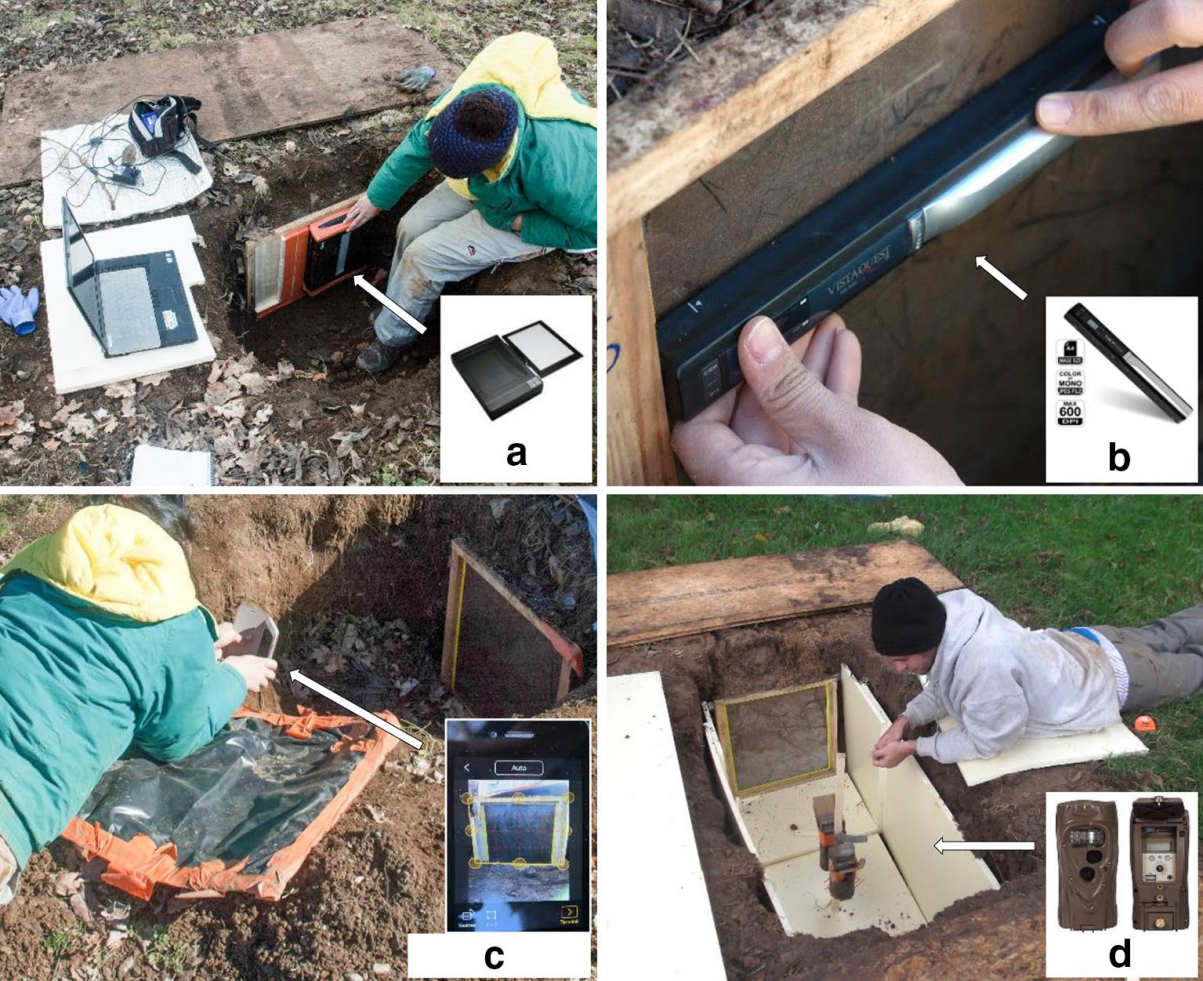
Four different methods were used to take images of walnut tree roots in rhizotrons: **a** flatbed scanner, **b** handheld scanner, **c** smartphone scanning application and **d** time-lapse camera. In (**d**), the time-lapse camera and rhizotron were placed into insulated boxes so that variations in temperature did not affect root growth. In the other rhizotrons, insulating material was placed over the rhizotron Plexiglas pane and removed before scanning

There are two common types of flatbed scanner, the CIS (Contact Image Sensor) and the CCD (Charge Coupled Device) scanners. A CIS scanner is more compact and requires less power than a CCD scanners and can usually run off battery power or the power from a USB port. CCD scanners, however, provide higher-resolution scans and are capable of scanning with a good depth of field. Accordingly, we used an Epson Perfection V370, high optical resolution of 4800 dpi and CCD technology that relies on a system of mirrors and lenses to project the scanned image onto the arrays. The lid of the scanner can be removed and the scanner connected to the computer via a USB cable and to a 15 V external battery (Fig. 1). The scanner can be placed horizontally or vertically. Four horizontal scans and a resolution of 300 dpi are needed for one 50 × 50 cm rhizotron.

#### Handheld scanner

Scanners are lightweight (Fig. 1) and portable. We used a Vista Quest HS-500 (USA) to take images at 300 dpi. Scans were taken by moving the scanner manually downwards on the surface of the rhizotron window. Three A4 (21 cm wide and 45 cm long) images are needed for one 50 × 50 cm surface in order to include the borders of our rhizotrons (see section on “Rhizotron installation”). The images can be saved on a micro Secure Digital memory card and the scanner requires only two AA alkaline batteries to function.

#### Manual tracing

If no electronic devices are available in the field, roots can be drawn manually with permanent color pens onto a transparent sheet placed over the rhizotron window. Colors indicate different observation times and the date of the observation is noted on the transparent sheet. Transparent sheets are then scanned in the laboratory using e.g. a scanner at a resolution of 300 dpi. (Sharp MX-3640 N PCL6, Canada). The manual tracing technique is not usually adequate for measuring root diameter precisely, because root diameter is not known, therefore it is not possible to select a pen with the appropriate point thickness. Nevertheless, manual tracing can be suitable for giving an estimate of root diameter class (e.g. Mao et al. [21]). In this study, we visually estimated root thickness and tried to use pens with the correct point thickness for tracing roots, so that we could compare results with those from the other methods.

#### Smartphone scanner application

**Fig. 2:**
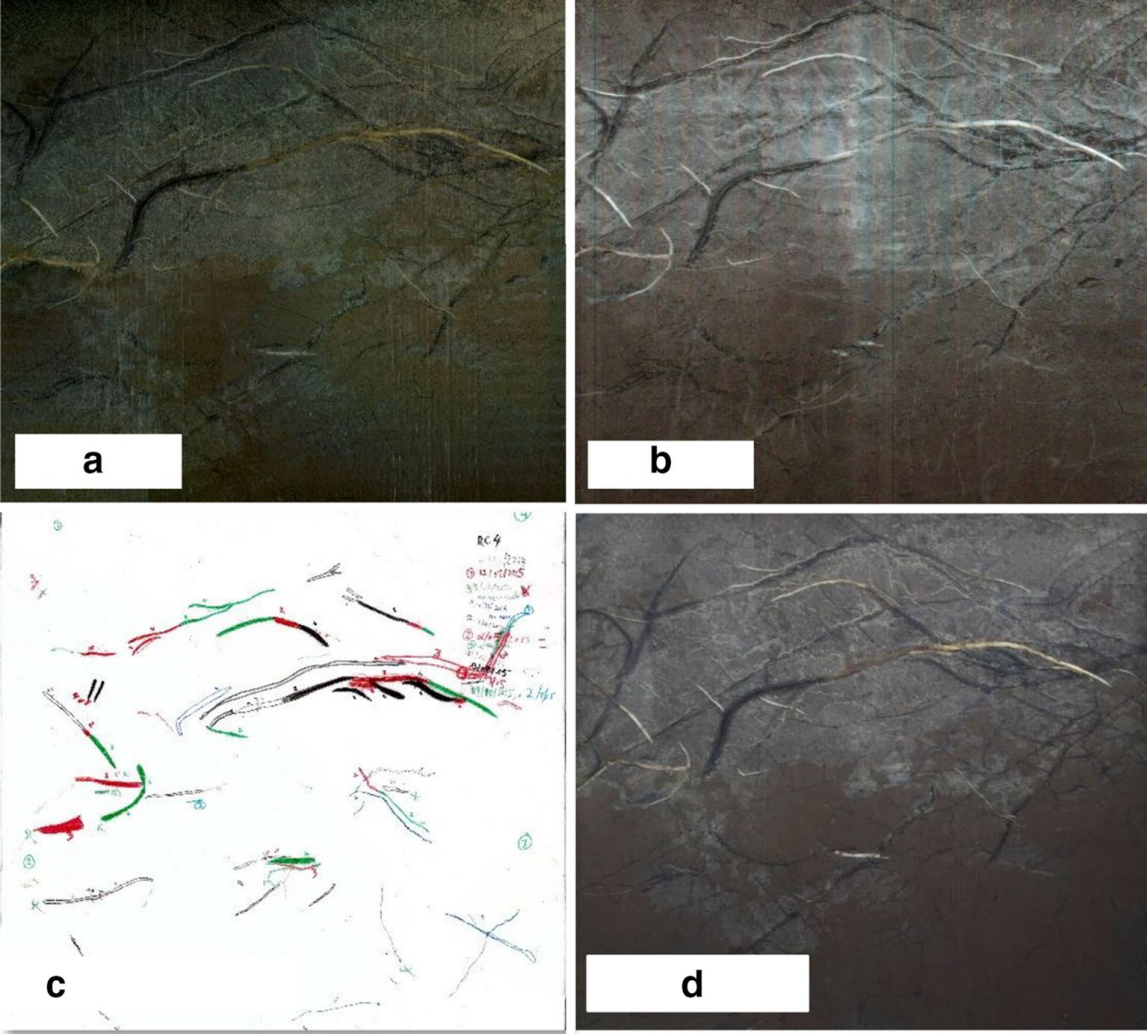
Examples of images taken by **a** flatbed scanner, **b** handheld scanner, **c** manual tracing on a transparent sheet and **d** smartphone scanner in the same rhizotron at the same date

To our knowledge, smartphone scanners have not yet been used for imaging root system growth. We took images using a scanning application on a smartphone (CamScanner INTSIG Information Co., Ltd, Shanghai, China) (version 3.9.5). The CamScanner application automatically detects object borders and removes background noise using image-processing technologies. This software adjusts image details, brightness and contrast and can return processed data in a JPG or PDF format. We also compared several generations of smartphone (iphone6, iphone4, and CAT^®^ S40) to compare the performance of the smartphone technology. To use the application on a rhizotron in the field, the smartphone must be held at a given distance (68 cm in our case) and a fixed scale (tape measure) must be scanned simultaneously to calibrate the scan (Fig. 2).

#### Time-lapse camera

Although often used to monitor the aerial phenology of vegetation, to our knowledge, time-lapse cameras have not yet been used for automated measurements of root growth and phenology in situ. Time-lapse cameras take photographs at regular intervals, determined by the user beforehand. We tested a Cuddeback Attack (U.S.A.) time-lapse camera with flash that takes photographs in color using LED bulbs (Fig. 1). Each camera was placed on a wooden cleat at a distance of 90 cm from the rhizotron. Photographs can be taken every 30 s (in our case, we took one photograph at 2 a.m. and at 12 h intervals thereafter). Time-lapse cameras can run for several months on an Alkaline battery (C (LR14) 1.5 V) without any human intervention.

### Comparison of methods

#### Test 1: previously scanned and measured root systems

**Fig. 3:**
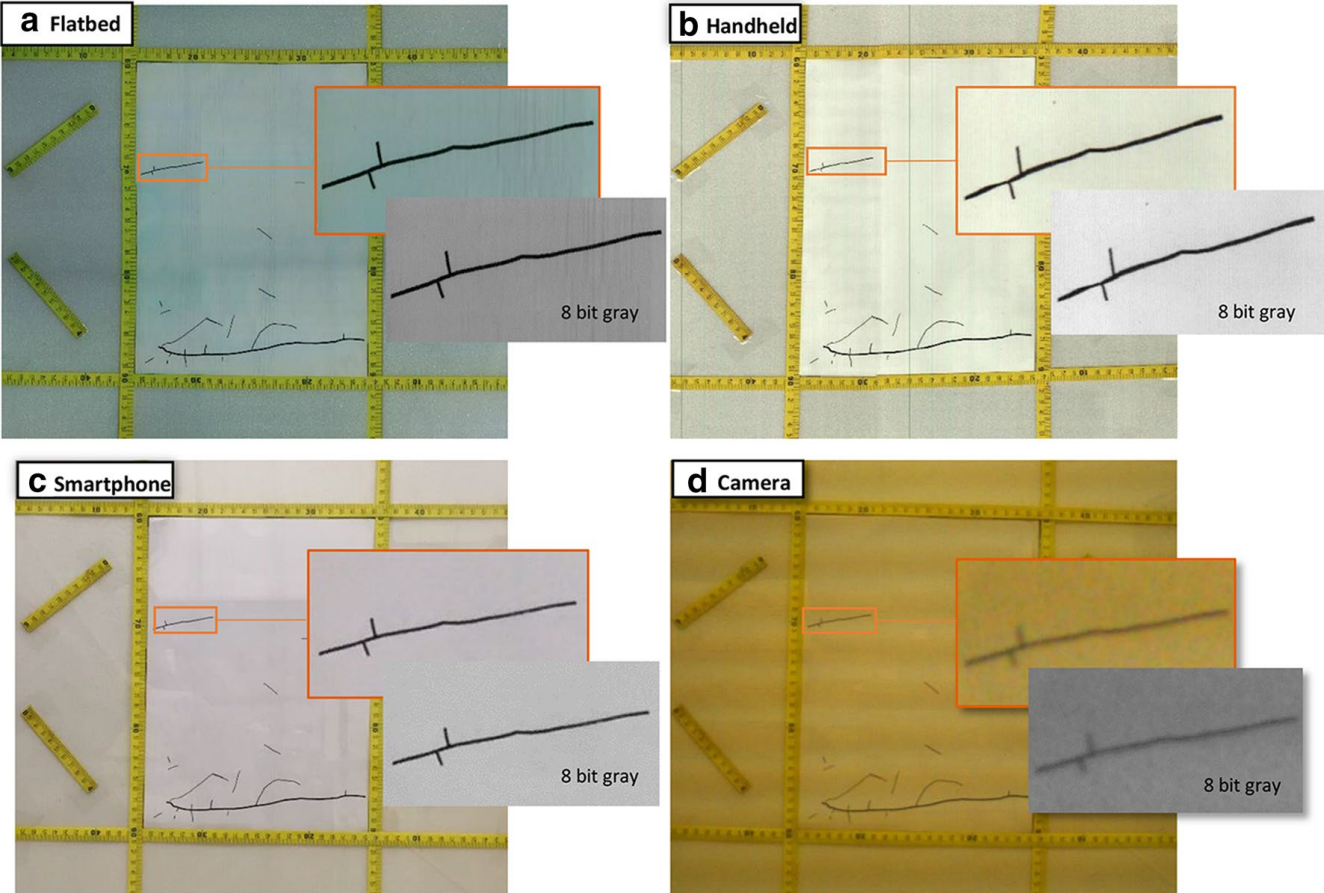
We tested the accuracy of **a** flatbed scanner, **b** handheld scanner, **c** smartphone scanner and **d** time-lapse camera by measuring root systems of known dimensions in the laboratory. A zoom of one root shows the quality of the images taken using each method before and after transforming the image to an 8 bit gray scale

To allow for a fully comprehensive comparison of data between scanning, manual and automated methods, we tested each method on previously scanned and measured root system (n = 35), and likewise on a measuring tape placed in different positions (Fig. 3) of known dimensions in a rhizotron (50 × 50 cm). The scanned root systems were measured using four methods: flatbed scanner, handheld scanner, smartphone scanners and the time-lapse camera. Data were imported to the SmartRoot software.

#### Test 2: Measurements in rhizotrons using scanners and manual tracing

We performed measurements at Le Beil, Madic, in the Cantal region, France (45°22′7.95″N, 2°28′1.46″E) (see section on study site for more details). We started the observations in October 2014 and fine root growth was measured every month from April to June 2015 using four methods: flatbed scanner, handheld scanner, smartphone scanner (iphone4) and the manual tracing (n = 25). Walnut fine roots are quite thick and so roots ≤ 4 mm in diameter were classed as fine roots.

#### Test 3: Measurements in rhizotrons using a time-lapse camera

A third set of measurements was performed at Cormont, in the Pas de Calais region, France (50°33′27.87″N, 1°44′3.08″E), (see section on study site for more details). Root growth was monitored in six rhizotrons twice a day from May to September. We focused our study on 21 roots growing over a period of 10 days for un easier understanding and comparison of results.

### Image analysis

Once images of root growth had been acquired, we conducted analyses of images using the semi-automated SmartRoot software [36]. SmartRoot is an operating system independent freeware based on ImageJ and uses cross-platform standards (RSML, SQL, and Java) for communication with data analysis softwares [36, 40]. Before analyzing roots with SmartRoot, when necessary, images need “stitching” together (e.g. with Adobe Photoshop CS3 software), if several have been taken (when the rhizotron surface area was greater than the field of the scanner). In our case, we transformed all images to 8 bit gray scale and then inverted them using ImageJ software so that roots were darker than the background of the image. The length and diameter of each root produced during one interval time (i.e. one month) were calculated for each rhizotron. Before analyzing a new sequence of images, SmartRoot provides the user with an icon to import the traces of the same roots from the previous image data file to superimpose them on this new image, which helps root elongation. This preceding image also helps determine whether the root is live (usually cream in color) or in a phase of senescence (shriveled, transparent or turning black) [3, 41–44]. We declared a root dead when it became completely black in color.

### Study sites

We measured fine root growth in situ in two agroforests. One was located at Le Beil, Madic, in the Cantal region, France (45°22′7.95″N, 2°28′1.46″E) at an elevation of 530 m, hereafter termed ‘continental’ climate. The agroforest comprised three transplanted tree species: hybrid walnut (*Juglans major (MJ209)* × *Juglans regia* L.), cherry (*Prunus avium* L.), sycamore maple (*Acer pseudoplatanus* L.) at 12 m × 8 m tree spacing and intercropped with permanent pasture (ovine or bovine pasture). All national guidelines and legislation were complied with when using these cultivars. Mean diameter at breast height (DBH) of all walnut trees at the site was 0.20 ± 0.02 m and mean height was 12.09 ± 1.30 m. Data are mean ± standard error. All trees were planted in 1994 at a density of 100 trees ha^−1^. Hybrid walnut at this study site starts leafing in early May and shedding in mid-November. The climate is continental with a mean annual temperature of 9.95 °C and a mean annual rainfall of 1174 mm (Météo France). The soil is silty and not deep, attaining an average maximum depth to bedrock of approximately 110 cm, on a 5°–10° slope.

The second agroforest was located at Cormont, in the Pas de Calais region, France (50°33′27.87″N, 1°44′3.08″E), hereafter termed ‘oceanic’ climate. The site is at an altitude of 40 m. The climate is oceanic, with a mean annual temperature of 11 °C and a mean annual rainfall of 777.9 mm (Météo France). Tree species comprised hybrid walnut (*Juglans nigra* × *regia* L.) and Maple (*Acer laurinum* L.) at 13 × 7.5 m tree spacing intercropped permanent pasture (ovine pasture). All trees were planted in 1999. The soil is silty clay and <2.5 m deep. The site is next to La Dordonne River. Mean DBH of walnut trees at the site was 0.30 ± 0.03 m and mean height was 14.75 ± 3.50 m. Hybrid walnut at this site starts leafing in early May and shedding in mid-November.

### Rhizotron installation

In the continental agroforest (Madic), we dug eight (1 m × 1 m × 1 m) trenches by hand in three rows of trees. Each trench was at a distance of 2 m from the nearest tree stem. Eight rhizotrons, or root windows (50 cm long × 50 cm wide × 0.5 cm thick), were installed. In the oceanic agroforest (Cormont), soil was deep (4 m) and comprised four (2 m long × 1 m wide × 2 m depth) trenches in one row of trees placed at 2 m from the nearest tree stem. One rhizotron was installed on two opposing faces of the trench (n = 8 rhizotrons in total). All rhizotrons were placed vertically at an angle of 15° from the face of the profile. This angle will permit the roots to grow downwards due to positive geotropism [21, 41]. Where the rhizotrons were to be placed on the trench, we gently removed the soil to make a flat surface and cut all roots on the profile with secateurs. The soil removed during the digging of the trenches was kept aside, and then sieved through a 5 mm size sieve and air-dried for several hours. The sieved and air-dried soil was then poured into the space between the window and the soil profile and slowly compacted using a wooden plank. Each rhizotron was covered with foil backed felt insulation and black plastic sheeting to protect roots from light and air temperature variations. All trenches were then covered with wooden boards and corrugated plastic to avoid damage from passing animals and to prevent direct rainfall and sunlight onto the rhizotrons. In the first three months after installation, no root growth was recorded to avoid over estimations of root growth [17].

### Root indicator calculation

We used the following method to estimate root elongation rate: individual root growth was evaluated by calculating the difference between the root length at *t−1* and at *t*. To determine the daily root elongation rate (RER), the mean of all individual root lengths produced between time *t* and *t−1* was divided by the duration of the corresponding period [3]. According to the literature, the characterization of dead roots is not obvious, particularly behind a transparent window [42]. We considered a root as live when it had a cream color and dead when it had turned black with no growth between two or more successive sessions until the last observation date occurred [3].

The equation we used to calculate RER was:

$$RER_{{{{\textit{t}} - 1}} ,\; {\textit{t}}} = \frac{{len{}_{\textit{t}} - len{}_{{\textit{t}} - 1}}}{{{\textit{P}}_{ {\textit{t-1}, \; \textit{t}} }}}$$

where, *RER*_*t−1, t*_ is the daily root elongation rate (in mm/day) from inventory time *t-1* to *t*; *l* *e* *n*_*t−1*_ and *l* *e* *n*_*t*_ are the lengths of the root n at inventory time *t*_*−1*_ and* t*, respectively; *p*_*t*__−1_, *t* is the period in days between inventory time *t*_*−1*_ and *t*.

At the oceanic site, as we took two photos per day (using the time-lapse camera), we aimed at testing whether our method could be used to estimate differences in RER during the day and at night [38]. Root elongation during 12 h during the daytime and night was calculated using:

$$\begin{array}{*{20}l} {RE_{\textit{day}} = len{}_{{{\textit{tn}}1}} - len{}_{{{\textit{td}}1}} } \hfill \\ {RE_{\textit{night}} = len{}_{{{\textit{td}}2}} - len{}_{{{\textit{tn}}1}} } \hfill \\ \end{array}$$

where* RE*_day_ and *RE*_night_ are root elongation lengths (in mm) during 12 h during daytime and night, repectively; *len*_*td*1_, *len*_*tn*1_ and *len*_*td*2_ are the lengths of a root successively observed at daytime 1 (2 pm), night time (2 am) and daytime 2 (2 pm), respectively.

### Semi-quantitative scoring decision matrix

Three criteria were taken into account to evaluate the five methods: (i) accuracy (image quality and resolution, deformation and contrast), (ii) effectiveness (time, expenditure and labour) and (iii) adaptability (ease of use in field and necessity of accessories).

### Statistical analysis

Root length and diameter obtained using each method were correlated with the previously scanned and measured root systems, to determine which method gave the best fit. Similarly, results from different generations of smartphones were compared. We then calculated relative values for cumulative length, mean diameter and RER, with regard to the flatbed scanner (reference value), which we assumed gave the most accurate dimensions [30, 32]. To calculate the relative value, we divided the value obtained for individual roots (using each method) by that obtained using the flatbed scanner.

A Shapiro–Wilk test was performed before each test to ensure if the investigated indicator followed a normal or non-normal distribution. Homogeneity of variances was checked. For data not normally distributed, analyses were followed by a Kruskal–Wallis Test for each factor. A post hoc analysis between root diameters was performed using the Nemenyi test of Kruskal–Wallis at *p* < 0.05 to determine which levels of the independent variable differ from every other level. All analyses were performed using R software, Version 2.15.3 (R Development Core Team 2013) at a significance level of <0.05.

## Results

### Test 1: Previously scanned and measured root systems

**Fig. 4:**
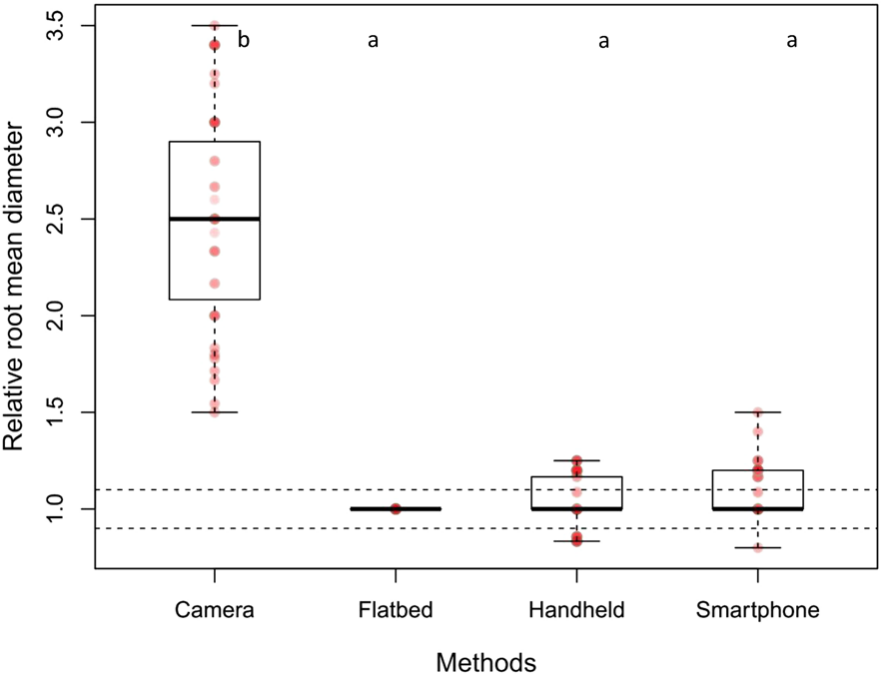
Comparison of the relative mean diameter (mm) of roots from root systems of known dimensions. The time-lapse camera significantly overestimated the diameter of roots compared to the three scanning methods (*p* < 0.001). Each *circle* represents diameter data for one root. *Differences in shading* intensity of *circles* indicate that one or more data points are superimposed. The *lower edge of the box* corresponds to the 25th percentile (Q1) data point, while the *top edge of the box* corresponds to the 75th percentile data point (Q3). The *line within the box* represents the median and the *whiskers* indicate the range of the data. The *two horizontal dashed lines* represent an interval of 10% above and below the median of the reference method (flatbed scanner). Different *letters above the boxplots* indicate statistically significant differences (*p* < 0.05) between methods

When images from the different generations of smartphone were compared, no significant differences were found with regard to root length and diameter between any models. When all methods (except for manual tracing) were compared together, there were no significant differences in the relative cumulative length of previously scanned and measured root systems. However, the time-lapse camera significantly overestimated the relative mean diameter of previously scanned and measured root systems compared to the other three methods (*p* < 0.001, Fig. 4). Although our time-lapse cameras had a high resolution (20 megapixels), this overestimation was likely due to the low optical resolution leading to a poorer quality of image. The SmartRoot software estimates the diameter of the root by diagonally measuring nodes along each root, but if the image is of low quality, SmartRoot will not be able to detect and distinguish correctly the border of the root (Fig. 3).

### Test 2: Measurements in rhizotrons using scanners and manual tracing

**Fig. 5:**
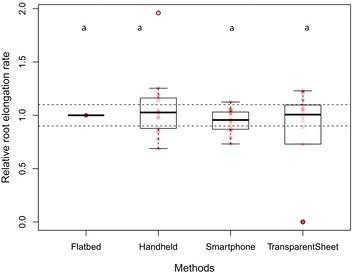
Comparison of the relative root elongation rate (RER) between the different image acquisition methods. Each *circle* represents RER for one root. *Differences in shading* intensity of circles indicate that one or more data points are superimposed. There were no significant differences in RER between the four methods. Each *circle* represents diameter data for one root. *Differences in shading* intensity of *circles* indicate that one or more data points are superimposed. The *lower edge of the box* corresponds to the 25th percentile (Q1) data point, while the *top edge of the box* corresponds to the 75th percentile data point (Q3). The *line within the box* represents the median and the *whiskers* indicate the range of the data. The *two horizontal dashed lines* represent an interval of 10% above and below the median of the reference method (flatbed scanner). *Different letters above the boxplots* indicate statistically significant differences (*p* < 0.05) between methods

**Fig. 6:**
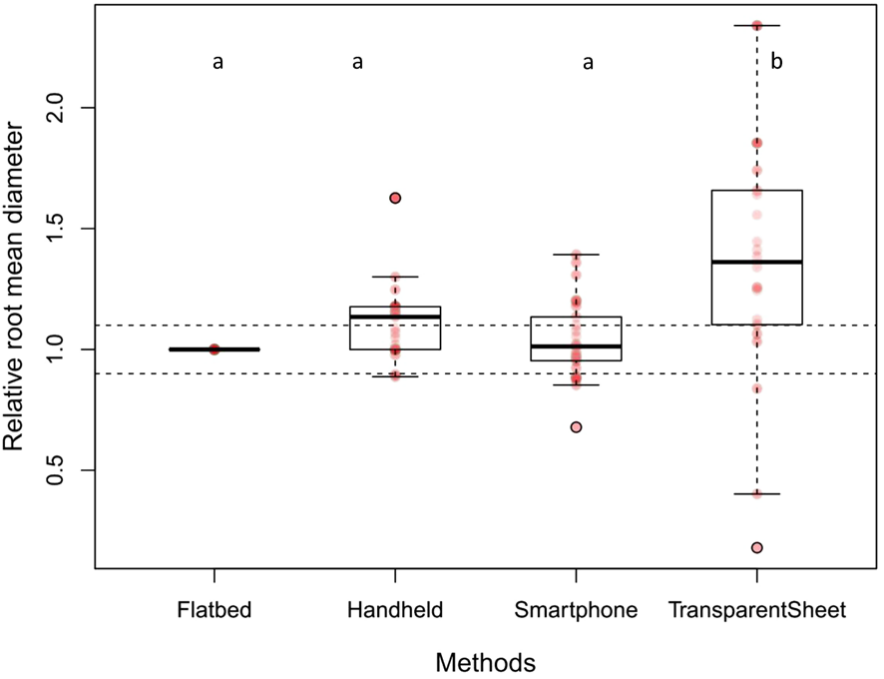
Comparison of the relative mean diameter (mm) of roots growing in rhizotrons in situ. Manual drawing on transparent sheets significantly overestimated the mean diameter of roots compared to the three scanning methods (*p* < 0.001). Each *circle* represents diameter data for one root. *Differences in shading* intensity of *circles* indicate that one or more data points are superimposed. The *lower edge of the box* corresponds to the 25th percentile (Q1) data point, while the *top edge of the box* corresponds to the 75th percentile data point (Q3). The *line within the box* represents the median and the *whiskers* indicate the range of the data. The *two horizontal dashed lines* represent an interval of 10% above and below the median of the reference method (flatbed scanner). *Different letters above the boxplots* indicate statistically significant differences (*p* < 0.05) between methods

There were no significant differences in relative RER between the four scanning methods (Fig. 5). However, manual tracing on transparent sheets significantly overestimated the relative mean diameter of roots (*p* < 0.001, Fig. 6). This overestimation was due partly to human error, as the Plexiglas window and the plastic sheet resulting in multiple layers, obscuring the root outlines, as well as the pens being either too thick or too fine for matching root diameter exactly.

### Test 3: Measurements in rhizotrons using a time-lapse camera

**Fig. 7:**
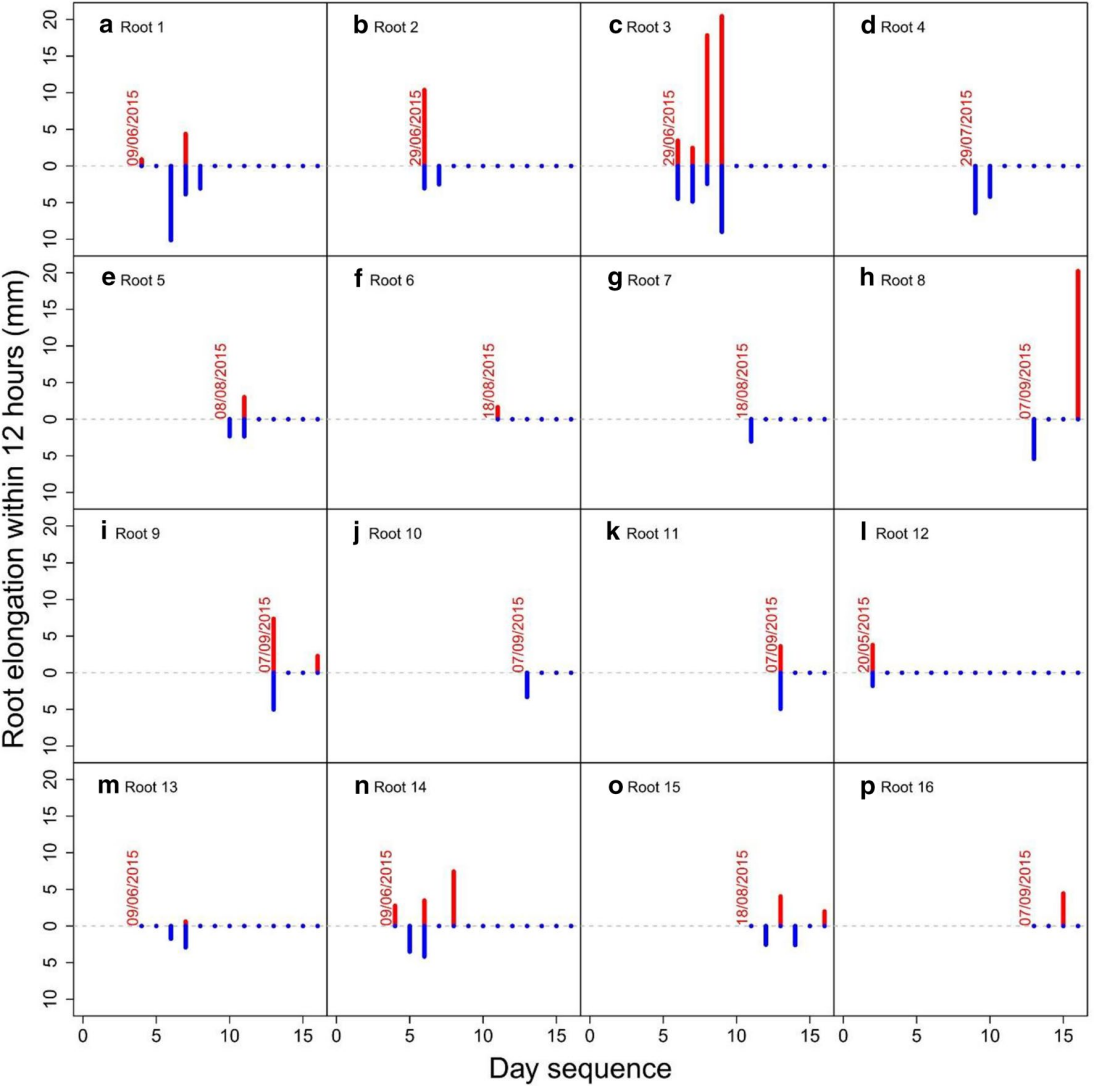
Root elongation reached 20 mm day^−1^ in certain roots. No significant differences were found in elongation between day and night over an interval of 10 days measured using a time-lapse camera. The *red color* represents root elongation during the day (data above the 0 on y axis) and the *blue color* represents root elongation during the night (data below the 0 on y axis). If no *bars* are present, roots did not grow during that period (even though they were still alive). The *date above the first data point* indicates when root growth started

**Fig. 8:**
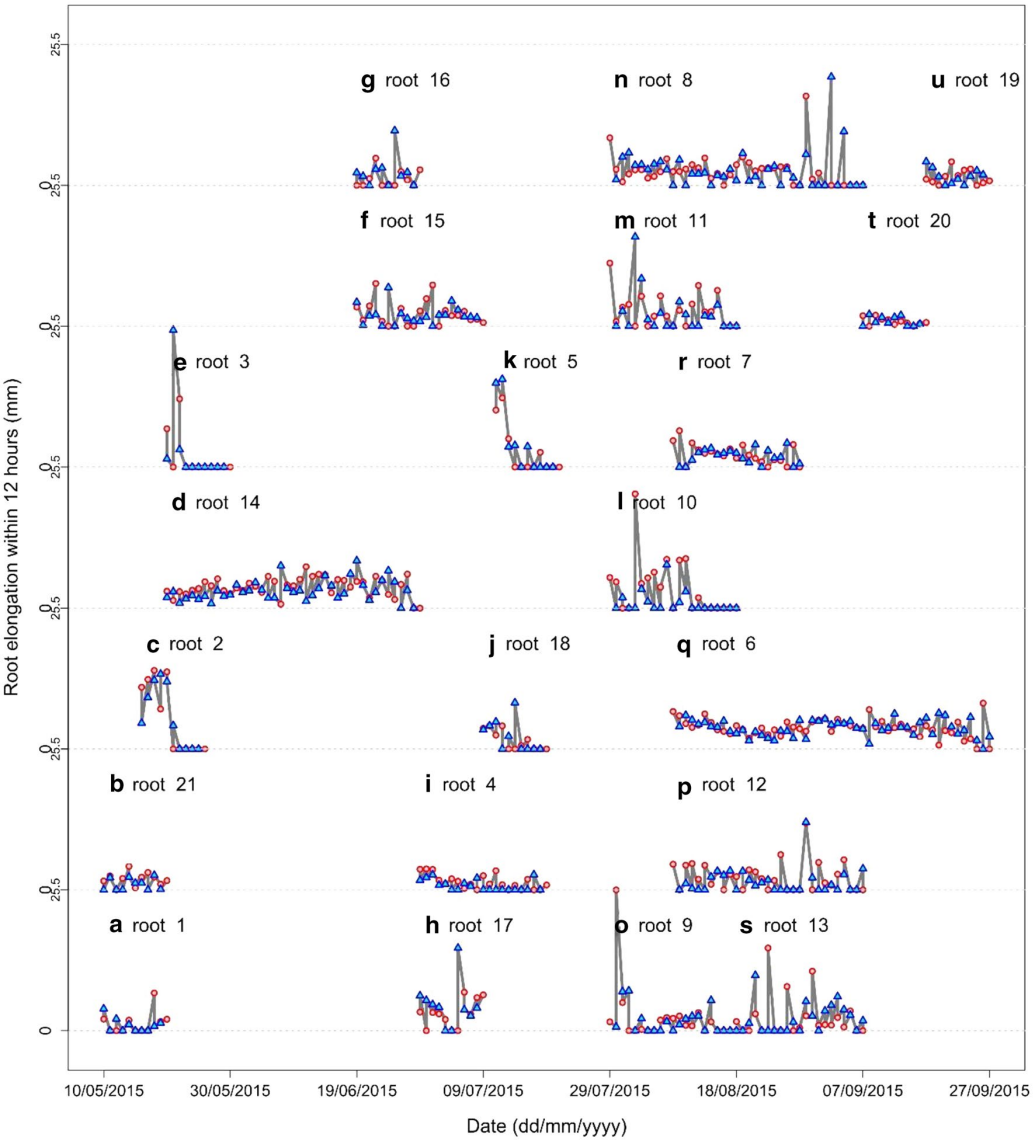
Root elongation (mm) in the daytime and at night, estimated using a time-lapse camera. Symbols: *circles* root elongation during the day, *triangles* root elongation during the night. For (*a*–*s*), each graph represents the elongation rate of one root randomly selected from a rhizotron throughout its entire lifespan

Some individual roots were found to elongate up to 20 mm in a single day (Fig. 7) and when mean elongation was cumulated over a period of 10 days, up to 48 mm of growth occurred (Fig. 8). When comparing root elongation between day and night (with a period of 12 h between the two measurements) no overall significant differences were found over the 10 day period examined (Fig. 7) or over the whole lifetime of individual roots (Fig. 8). This method therefore also allowed us to estimate differences in root elongation between day and night.

## Discussion

Studies on root growth have been numerous over the last decade and a significant progress in evaluating root morphology has been made. However, research remains challenging and costly especially in the natural environment. Many nondestructive methods, such as rhizotrons [18–21] and minirhizotrons [3, 22, 23] have been developed to overcome some of the limitations of observing root systems in the natural environment and to offer direct and repeated observations of root system morphology. Image quality obtained from rhizotrons and minirhizotrons is crucial for an accurate quantification of root growth through image analysis.

We showed that all five methods for imaging root systems can be used to determine root length, but that if accurate measurements of root diameter are required, a scanner must be used, and not the time-lapse camera nor manual tracing. The smartphone scanning application was found to be perform best overall when considering image quality. Images were sharply contrasted, of high resolution and deformation was minimal. The application was free for smartphones and did not need any accessories. Only a short amount of time was spent in the field acquiring data and image treatment can be carried out directly in the application (Table 1). Another advantage of the smartphone is its genericity and wide community of developers and any of the models tested could be used, with no consequences for results obtained. Many third party hardwares (such as additional lenses, holders, batteries) and software tools (automated cloud backup, automated geographical tagging, etc.) are available, often at minimal cost. It should also be noted that the quality of the smartphone camera and the lifetime of batteries have been constantly improved by manufacturers, probably at a much faster rate than for specialized equipment. In contrast, the flatbed scanner has many accessories so it is not easily transportable and needs four images for one 50 × 50 cm rhizotron, therefore much time is needed in the laboratory to merge images before analyzing them. Additionally, automatic flatbed scanners have not been developed yet. Thus, the scanner cannot automatically acquire images in the field over a long period because of the need for a power supply in the field. However, the method was rapid, easy to use and inexpensive. Image quality was very high as also found by other users [30, 32, 45] (Table 1). Likewise, the advantage of the handheld scanner is that it is quick, portable and the images are of good quality [37]. Three images were needed for one 50 × 50 cm surface with this method, and the major constraint with this scanner is the size of images (29.7 × 21.0 cm), so more time is needed for merging images manually before analyzing them (Table 1). The distinct advantage of tracing onto transparent sheets is their inexpensive price (Table 1), but inaccuracies due to human error and optical effects occur, resulting in an overestimation of root diameter. As scanning methods are not yet automated for use in the field, the main advantage of the time-lapse camera is that it can be left in place for several months without any manual intervention and it is relatively inexpensive (Table 1). However, the quality of images taken with the time-lapse cameras was poor and a certain amount of reflection occurs due to the flash, leading to an ultimate overestimation of root diameter. The low quality of images taken is because the lenses have less optical resolution compared to e.g. a smartphone camera, resulting in blurred photos. The optical resolution represents the physical resolution to resolve detail in the object that is being imaged via an imaging system. Smartphone cameras and digital cameras have been developed to be capable of defining the smallest discernible detail in an image, resulting in a better spatial resolution which states the clarity of an image and this resolution refers to the number of pixels used to construct the image. This spatial resolution depends on properties of the system creating the image, not just the pixel resolution (pixels per inch). For example, in our study, although the smartphone had less resolution (5 megapixels) than the time-lapse camera (20 megapixels), it produced a better quality of image. This quality is because the smartphone imaging system can detect spatial differences and this spatial resolution can be influenced by diffraction, aberrations, imperfect focus, lens, size of the sensor and other imaging system components. Furthermore, as the automated time-lapse camera was programmed to autofocus mode, the camera may focus on the wrong part of the image depending on the environment where the image is captured. We successfully used the time-lapse camera to compare root elongation during the day and at night, but no significant differences were found. Nevertheless, from Figs. 7 and 8, it can be seen that some roots grow mostly at night and others grow mostly during the day, although the reason for this disparity between roots is not known.

**Table 1:** Multiple criteria evaluation/semi-quantitative scoring decision matrix

Method	Smartphone application	Flatbed scanner	Handheld scanner	Time-lapse camera	Transparent sheet
Image quality	*** High	*** High	*** High	* Low	–
Deformation	***	***	***	**	**
Length	No	No	No	No	No
Diameter	No	No	No	Yes	Yes
Time taken	****	**	***	****	*
For 1 rhizotron	40 s	12 min	1 min	0 s	12 min
For 8 rhizotrons	7 min	96 min	16 min		95 min
Cost	** Phone100–700€ Apps free	** 150 €	** 80 €	* 190 €/rhizotron	****
Usage in field	**** Easy/1 person	** Difficult/2 persons	*** Easy/1 person	*** Autonomous	* Difficult/1 person
Accessories	*** No	* Yes/battery and PC	*** No	*** No	*** No
Time between visits	** 2–4 weeks	** 2–4 weeks	** 2–4 weeks	*** 4–5 months	** 2–4 weeks
Time to treat images before analysis	*** 0 s	* 3.30 min/image	* 4 min/image	* 3 min/image	*** 0 s

**** Optimal, *** good, ** fair, * poor, – not applicable

To the best of our knowledge, the smartphone scanning application and an automated time-lapse camera have never been used to measure root growth in the field. Both methods are inexpensive and easy to use, especially compared to more sophisticated techniques such as minirhizotron scanners. The advantage of rhizotrons over minirhizotrons is that the above variety of inexpensive techniques exist worldwide for quantifying root growth in the field. The equipment needed to observe and record color video pictures of roots in minirhizotrons [9, 46] is commercially available at a cost of approximately 10,000 euros for one circular scanner [3] or one camera video. Additionally, the field of vision in a minirhizotron is small (20 × 20 cm) and is not suitable for heterogeneous forest stands, where the spatial position of roots of different diameter classes can lead to root-free patches in the soil. The 50 × 50 cm rhizotrons we used in our study enable more tree roots to be captured in one image, thus increasing statistical robustness.

## Conclusion

We tested several methods for monitoring root growth and acquiring images in rhizotrons in the field. Our results show that scanners and time-lapse cameras provide correct measurements of root growth and length in the field but users should be aware of possible artifacts. Time-lapse cameras overestimate root diameter but are useful for taking frequent images of root elongation in the field over several months, without any manual intervention. Taking into account image accuracy, time spent and cost, we found the smartphone scanner to be the optimal method for monitoring root growth in the field. Future generations of smartphones could scan images and transfer data automatically, with a minimum of human intervention, thus improving the methodology. Likewise, the development of digital time-lapse cameras with a higher optical resolution, or similar to the optical resolution of smartphones, should also be undertaken.

## Abbreviations

RER: root elongation rate
RE: root elongation
